# Dr. Peter M. Wise

**Published:** 1907-10

**Authors:** 


### OBITUARY.

---

Dr. Peter M. Wise, of New York, died at the J. Hood Wright Hospital in that city, September 22, 1907. He was a native of Clarence, Erie County, N. Y., and a graduate of the Medical Department of the University of Buffalo in 1872. Early in his medical career he turned his attention to nervous and mental diseases, especially insanity, and became an assistant at the Willard State Hospital. After the Ogdensburg State Hospital was built Dr. Wise was appointed its first superintendent and while there published a book relating to the care of the insane.

Dr. Wise was appointed chairman of the State Lunacy Commission by Governor Morton, whereupon he vacated his position at Ogdensburg and removed to Albany. Finally, he retired from public service and then went to New York, engaging in private practice. Some years ago he began to suffer from locomotor ataxia, which caused increasing distress and pain, notwithstanding strenuous efforts at treatment. A year or more ago, according to press dispatches, he prepared a mixture of sedatives which gave him relief for a time, though increasing doses were necessary of late, and it is supposed his death was caused by a double dose of the remedy taken to relieve a paroxysm of pain.

